# Safety Profile and Effects of Pulsed Methylprednisolone on Vital Signs in Thyroid Eye Disease

**DOI:** 10.1155/2015/457123

**Published:** 2015-11-22

**Authors:** Kai-Ling Yong, Chiaw Ling Chng, Hla Myint Htoon, Lee Hooi Lim, Lay Leng Seah

**Affiliations:** ^1^Singapore National Eye Centre, 11 Third Hospital Avenue, Singapore 168751; ^2^Singapore Eye Research Institute, 11 Third Hospital Avenue, Singapore 168751; ^3^Singapore General Hospital, Outram Road, Singapore 169608; ^4^Duke-NUS Graduate Medical School, 8 College Road, Singapore 169857; ^5^Yong Loo Lin School of Medicine, National University of Singapore, 1E Kent Ridge Road, NUHS Tower Block, Level 11, Singapore 119228

## Abstract

*Objective*. To analyze changes in vital signs (heart rate (HR), systolic (SBP), and diastolic blood pressure (DBP)) during and after intravenous methylprednisolone (IVMP) and any other adverse effects.* Methods*. Retrospective review of charts of patients who received IVMP as treatment regime for thyroid eye disease. All subjects had vital signs charted during and after infusions.* Results*. This study included 38 subjects and a total of 242 infusions administered. IVMP resulted in a small but significant percentage drop in mean SBP at 30 min (*p* < 0.001) and 60 min (*p* = 0.03) but no difference at 90 min. There was also small but significant percentage drop in mean DBP and HR (DBP: *p* < 0.001 for 30 min, *p* = 0.001 for 60 min, and *p* = 0.02 for 90 min and HR: *p* < 0.001 for 30 min, 60 min, and 90 min). There were no cumulative effects on change of blood pressure or HR. There were 6 episodes of bradycardia (2.5%) and 12 episodes of moderate to severe hypertension (5%). No significant cardiovascular or hepatic toxicity was found.* Conclusion*. IVMP is relatively safe and efficacious. IVMP demonstrated mild and noncumulative effects on vital signs. Severe hypertension may occur in susceptible individuals such as those with underlying hypertension and uncontrolled thyroid dysfunction, whereas bradycardia may be more likely in those on beta-blockers.

## 1. Introduction

Thyroid eye disease (TED) is an autoimmune process which involves abnormal proliferation and activation of orbital fibroblasts, increased glycosaminoglycans, and proinflammatory cytokine production. Cytokines can modulate the immune reaction in TED by increasing major histocompatibility complex class II, adhesion molecules, prostaglandin, and heat shock protein expression in the orbit [1, 2]. Glucocorticoid therapy is effective in TED as it has anti-inflammatory and immunosuppressive actions, inhibits release of immune mediators such as cytokines, interferes with function of T and B lymphocytes, and decreases glycosaminoglycan production by orbital fibroblast [3, 4].

Pulsed intravenous methylprednisolone (IVMP) therapy is an established treatment of active moderate to severe TED and sight-threatening dysthyroid optic neuropathy [5]. IVMP therapy is also more efficacious and associated with fewer side effects than oral steroids [6, 7]. The favourable response rates of pulsed IVMP are about 80%, as compared with about 60% for oral steroids [8].

However, there are concerns regarding the safety profile of pulsed IVMP, especially on the cardiovascular system. There are many known adverse effects of pulsed IVMP therapy. Minor effects include flushing, mild hypertension, gastritis, weight gain, depression, hyperglycemia, insomnia, palpitations, sinusitis, and urinary tract infections [7, 9–12]. Major adverse events reported include cardiovascular events, acute liver damage, and even death [13–18]. There is no established consensus on the monitoring of patients during and after IVMP administration. Previous studies had reported IVMP resulting in hypertension in TED patients [9, 19] or bradycardia [20, 21], but none looked at the extent and pattern of blood pressure and heart rate fluctuations at various time points after the steroid infusions. In addition, literature detailing the risk factors for such fluctuations is scarce [9, 21].

The purpose of this study is to analyze the changes in vital signs (including heart rate (HR), systolic (SBP), and diastolic blood pressure (DBP)) during and after IVMP infusion (0 min, 30 min, 60 min, and 90 min), as well as changes in electrocardiogram (ECG), potassium levels, glucose levels, and any other adverse effects.

## 2. Methods

We performed a retrospective review of the charts of TED patients who underwent pulsed methylprednisolone therapy from 2004 to 2010 in Singapore National Eye Centre. Numerous different parenteral regimes for IVMP have been reported, with no clear consensus on the optimum dosage, dosing intervals, and duration of treatment [8, 22]. The dosing regimen used in our institution during the study period consisted of 1 g hourly per day over 3 days given in the outpatient setting [11, 23, 24]. The indications of IVMP were active TED or compressive optic neuropathy. The study was carried out in accordance with the tenets of World Medical Association's Declaration of Helsinki.

All subjects were examined for best correctable visual acuity (BCVA), colour vision, VISA scoring, and slit-lamp examination prior to pulsed methylprednisolone therapy. An inflammatory score of 4 or more on VISA was considered as active TED [25]. Baseline blood investigations including full blood count, liver function test, renal panel, glucose, hepatitis B and hepatitis C screening, and ECG were performed. Patients who had any symptoms or signs of infection, severe gastric disease, unstable cardiac disease, active liver disease, and poorly controlled diabetes mellitus or hypertension were excluded from IVMP treatment.

All subjects had their vital signs (HR, SBP, and DBP) charted before infusion (0 min), at 30 min (during infusion) and 60 min (end of infusion), and after infusion (90 min). ECG and capillary glucose level were checked at 60 min. Serum potassium was measured before and after each infusion for all subjects before discharge. All subjects had to complete a questionnaire at the end of infusion which documented any subjective complications experienced.

Subjects were reviewed after each cycle of 3 doses of IVMP for BCVA, colour vision, VISA score, and full slit-lamp examination. Full blood count, liver function test, and renal panel were performed. Subjects were reviewed at interval of 1 to 3 months after treatment depending on their condition and physician's discretion. Subjects may undergo repeated cycles of IVMP if the TED was considered active or patients developed signs of compressive optic neuropathy. Subjects may also be given adjuvant methotrexate or radiotherapy after IVMP treatments were completed.

Statistical analysis was performed with SPSS software (IBM Corp., released 2010, IBM SPSS Statistics for Windows, Version 19.0, Armonk, NY). Parametric data was analyzed using paired *t*-test. A linear mixed model was utilized to analyze and account for the cumulative effect of the multiple doses of each cycle of methylprednisolone. Three candidate covariance structures were checked for minimizing the mean squared error of predictions, first-order autoregressive [AR(1)], compound symmetry (CS), and unstructured (UN), to estimate the fixed effects and we have selected the structure with the smallest Akaike's Information Criterion (AIC) and Bayesian's Information Criterion (BIC). Linear mixed models were fitted and compared using restricted maximum likelihood methods. A statistical significance level of 0.05 was used for this study.

## 3. Results

The study included 38 subjects who underwent a total of 242 infusions of IVMP (Table 1). The mean age (standard deviation, SD) was 48.4 ± 8.8 years and 65.8% were male. The subjects underwent a mean (SD) of 2.3 ± 1.5 cycles of IVMP (range: 1 to 7 cycles). The main indication of therapy was active TED (68.4%). Twenty-six subjects required adjuvant therapy of methotrexate, and 10 subjects required radiotherapy. Eight subjects went for subsequent decompression surgery. More than half (57.9%) of the subjects showed hyperthyroidism (defined as raised free thyroxine with a suppressed thyroid stimulating hormone) on thyroid function test at baseline.

**Figure 1:**
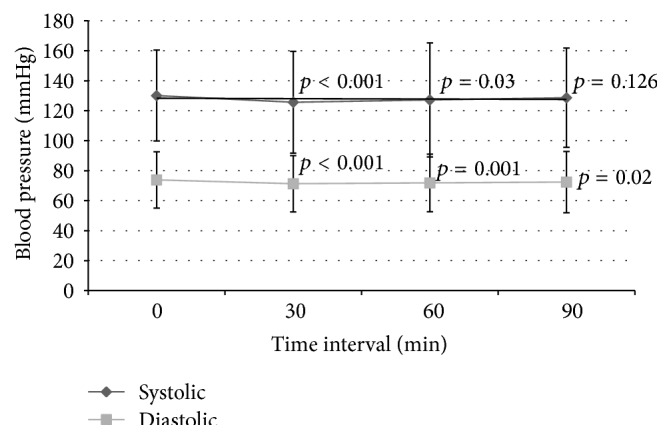
Systolic and diastolic blood pressure over time. Significance value pairwise comparison for SBP (paired *t*-test): *p* < 0.001 for 0 min versus 30 min, *p* = 0.03 for 0 min versus 60 min, and *p* = 0.126 for 0 min versus 90 min. Significance value pairwise comparison for DBP (paired *t*-test): *p* < 0.001 for 0 min versus 30 min, *p* = 0.001 for 0 min versus 60 min, and *p* = 0.02 for 0 min versus 90 min.

**Figure 2:**
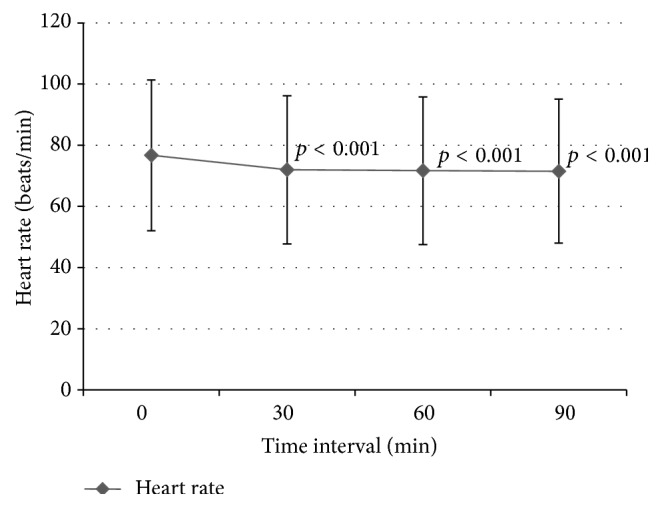
Heart rate over time. Significance value pairwise comparison for heart rate (paired *t*-test): *p* < 0.001 for 0 min versus 30 min, *p* < 0.001 for 0 min versus 60 min, and *p* < 0.001 for 0 min versus 90 min.

There was a small but statistically significant percentage drop in mean (SD) SBP at 30 min and 60 min compared to baseline SBP (−3.31 ± 9.91%, *p* < 0.001, and −2.25 ± 12.18%, *p* = 0.03, resp.) but no significant difference after stopping infusion at 90 min (−0.67 ± 11%, *p* = 0.126). There was also small but statistically significant drop in mean percentage change DBP at 30 min, 60 min, and 90 min compared to baseline DBP (−2.92 ± 10.6%, *p* < 0.001, −2.1 ± 11.82%, *p* = 0.001, and −1.34 ± 12.63%, *p* = 0.02, resp.) (Figure 1). The IVMP resulted in a significant decrease in mean percentage change HR (*p* < 0.001) from 0 min to 30 min (−5.73 ± 10.14%), 60 min (−5.58 ± 11.36%), and 90 min (−5.92 ± 12.12%) (Figure 2). After comparing the SBP, DBP, and HR between the 3 doses of IVMP, there were no statistically significant cumulative effects of methylprednisolone on percentage change of SBP, DBP, and HR between doses 1, 2, and 3 within each IVMP cycle.

There was a significant decrease (*p* = 0.045) in mean (SD) potassium levels from 4.14 ± 0.36 mmol/L to 4.08 ± 0.37 mmol/L, but all were within normal clinical range. There was no significant cumulative effect on potassium levels between each dose of the cycle (*p* = 0.837). The mean (SD) random capillary glucose level was significantly increased from 7.76 ± 3.28 mmol/g to 11.87 ± 5.87 mmol/g after infusion (*p* < 0.001). There was also no significant cumulative effect on glucose levels between each dose (*p* = 0.563). There were 4 patients (10.5%) who required subcutaneous insulin therapy to correct the hyperglycemia, and all these subjects had preexisting type 2 diabetes mellitus.

Overall, there was improvement in both BCVA and colour vision after treatment after each cycle of 3 doses of 1 g IVMP, although it was not statistically significant (*p* = 0.348 and *p* = 0.685 for BCVA right and left eye and *p* = 0.459 and *p* = 0.641 for colour vision right and left eye, resp.). However, if only the subjects with compressive optic neuropathy were analyzed, there was a significant improvement in both visual acuity and colour vision (Table 2). There was also significant improvement (*p* < 0.001) of VISA inflammatory score from mean score (SD) of 4.29 ± 1.14 before treatment to 1.92 ± 1.12 after treatment.

Examining the overall adverse effects of IVMP, there were 6 (2.5%) episodes of bradycardia (HR less than 50 bpm), 8 (3.3%) episodes of tachycardia (HR more than 100 bpm), and 12 (5%) episodes of moderate to severe hypertension or stage 2 hypertension (SBP > 160 mmHg or DBP > 100 mmHg) [26]. These 12 episodes occurred in 4 subjects; one of them was hyperthyroid with no underlying hypertension and two were hypothyroid and hypertensive while one was euthyroid with underlying hypertension. Three subjects (1.2%) had severe hypertension (as defined by SBP ≥ 180 mmHg or DBP ≥ 110 mmHg) (Table 3). All these subjects had documented normal blood pressure before initiation of IVMP. One patient developed urticarial rash and the pulsed methylprednisolone was stopped immediately and hence did not complete the cycle of 3 doses of therapy. The patient's vital signs remained stable. Two patients (5.3%) had mildly deranged liver enzymes, which all reverted to normal by 1 year after the administration of IVMP. No new arrhythmias or ischemic events were noted on ECG monitoring for all subjects. Two subjects complained of breathlessness and 1 had chest tightness, with no accompanying vital sign or ECG changes. These symptoms did not recur during the subsequent doses of IVMP in the two subjects.

## 4. Discussion

Pulsed methylprednisolone infusion for treatment of active TED has been shown to be relatively safe and effective. IVMP therapy is established to be effective in improving inflammation in active TED, more efficacious than oral steroids with less adverse events [8, 27]. Consistent with the current literature, our study showed that these patients had improved inflammatory score and those with compressive optic neuropathy had improvement in visual acuity.

The mechanisms by which steroids lead to hypertension may be multifactorial. Steroids result in sodium retention and volume expansion [28] and act on nitric oxide synthase pathway [29]. Steroids may also mediate vascular vasoconstrictor sensitivity to catecholamines and other vasoconstrictor hormones systems [30, 31]. Our results showed that IVMP had a general trend in lowering the SBP and DBP; however the mean change was small and may not be clinically significant. Although the effects of IVMP on BP were not cumulative in our study, we found 12 (5%) episodes of moderate to severe hypertension (SBP ≥ 160 mmHg or DBP ≥ 100 mmHg), and three subjects had SBP 180 mmHg or more at the end of infusion. All these patients' blood pressure returned to normal at 90 minutes and remained asymptomatic. Subsequent administration of IVMP did not cause such dramatic changes in blood pressure. Nonetheless, IVMP should be used with caution in those with poorly controlled hypertension since sudden fluctuation in blood pressure may still exert stress on the cardiac system and result in pulmonary oedema and acute heart failure [13].

There were three patients with thyroid dysfunction at baseline who developed moderate to severe hypertension during IVMP infusion. Both hyperthyroidism and hypothyroidism are associated with increased blood pressure; hypothyroidism is a recognised cause of secondary hypertension [32], and hyperthyroidism results in higher risk of elevated SBP, widened pulse pressure, sinus tachycardia, and atrial fibrillation and may even lead to heart failure [33–35].

IVMP administration has been associated with cardiac arrhythmias in some studies [20, 36]. Although IVMP resulted in decrease in HR that remained low at 90 min after IVMP infusion in our study, there was no cumulative effect on the subsequent dose the next day. All the subjects who developed bradycardia in our study were on beta-blockers, while the subjects who developed tachycardia were hyperthyroid and were already tachycardic at baseline. None of the patients developed atrial fibrillation. Our study suggests that patients on beta-blockers were more susceptible to developing bradycardia. Bradycardia after steroid administration had been reported, but the mechanisms were not well studied [20, 21]. One explanation for the occurrence of reversible bradycardia is by the pharmacokinetics of steroid operating through the hypothalamic-pituitary axis suppression [37].

IVMP may also cause hyperglycemia, especially in diabetic patients and transient deranged liver enzymes; thus careful screening of patient's glucose levels and liver function should be performed prior to IVMP administration [10, 38]. In our study, the four subjects who required insulin therapy for correction of hyperglycemia were all diabetics. Similarly in a large study by Feldman-Billard et al. [10], the authors found that methylprednisolone therapy resulted in raised glucose levels, but the effects were different in nondiabetics and diabetics. In nondiabetics, the hyperglycemia was maximum after first pulse and returned to baseline with subsequent pulses, but diabetic patients had cumulative effects of the hyperglycemia with repeated pulses, and a quarter of the diabetic patients required insulin. Our study showed that four out of seven diabetic patients required insulin at some point during their cycles but did not show any overall cumulative effects of glucose with repeated pulses. However, our study did not analyze the cumulative effects on diabetics alone due to the small numbers. Although the hyperglycemic effects may be transient and correctable, acute hyperglycemia is an independent risk factor of cardiovascular events and should be prevented [39, 40].

Hypertension and hyperglycemia may also result in pathophysiological changes in the retina, such as arteriolar narrowing, retinal haemorrhages, microaneurysms, exudates, and optic disc swelling [41, 42]. Although the hypertensive effects of methylprednisolone may be transient, the acute increase of blood pressure may lead to vascular necrosis, resulting in necrosis of smooth muscle and endothelial cells [43]. Acute retinopathy changes associated with hypertension may be generally reversible, but potential worsening of diabetes control induced by repeated pulsed high dose methylprednisolone therapy may exert irreversible damage to end target organs through the “metabolic memory” [43].

Marinó et al. estimated acute liver damage in 0.8% of TED patients who underwent IVMP [15]. They postulated that IVMP caused liver damage by either direct damage on liver cells, precipitation of virus-induced hepatitis, or reactivation of immune system after IVMP-induced immune suppression resulting in autoimmune hepatitis. The damage may be dose dependent [44]. Our subjects underwent a large range of IVMP courses (one to seven cycles), and seven patients had cumulative doses of more than 9 g. Of note, six of these subjects were treated between 2004 and 2007, which were before the recommendation that IVMP should not exceed cumulative dose of 8 g [17]. One subject had seven cycles administered in parts over three years. None of our patients developed liver failure, and the two subjects who had mild increase in liver enzymes (less than three times of the upper reference limit) had less than 8 g administered. Their liver function tests returned to normal within 1 year after the IVMP pulses were stopped. In our study, one subject developed urticarial rash near the end of the 60-minute infusion. Anaphylactic shock had been reported in IVMP administration [45, 46]; hence it is recommended that the drug be infused slowly over 60 min.

Despite demonstrating a decrease in potassium levels after IVMP infusion, all the subjects' potassium levels were within normal clinical range. This may suggest that in relatively well patients with normal potassium at baseline, there is no need to recheck their potassium levels after IVMP administration. The caveat is that these patients should not have any underlying medical conditions that may result in them having electrolyte abnormalities, such as renal failure or use of medications such as diuretics.

The main strengths of our study were the large number of 242 infusions analyzed and recorded patients who had multiple infusions administered with the long follow-up duration. The main limitations were the small number of study subjects and the large variance of dosage administered (cumulative dose: 1 g to 21 g) instead of a fixed cumulative dosage administered. However, such variation is expected in the management of patients with active TED where response to IVMP may be variable and other forms of second-line treatment may be required at different time points during the course of treatment.

## 5. Conclusion

IVMP is a relatively safe and efficacious therapy for active moderate to severe TED and optic neuropathy. Overall, IVMP administration resulted in mild and noncumulative effects on heart rate and blood pressure. However, patients with poorly controlled hypertension, diabetes, or active liver disease should have their underlying conditions optimised before undergoing pulsed methylprednisolone therapy. Severe hypertension may occur during IVMP treatment in susceptible individuals such as those with underlying hypertension and uncontrolled thyroid dysfunction, whereas bradycardia may be more likely in those on beta-blockers. Patients whose potassium levels are normal at baseline and are not predisposed to hypokalemia from drugs or medical conditions may not require close monitoring of their potassium levels after every dose.

## Figures and Tables

**Table 1:** Characteristics of study population.

	Overall (*N* = 38)
Mean age (SD), years	48.81 ± 8.8
Male (ratio)	25 (65.8%)
Mean number of cycles (SD)	2.34 ± 1.53
Range	1–7
Smoking status	
Current smokers	10 (26.3%)
Nonsmokers	24 (63.2%)
Ex-smokers	4 (10.5%)
Preexisting comorbidities	
Heart disease	3 (7.9%)
Hyperlipidemia	4 (10.5%)
Stroke	1 (3.2%)
Diabetes	7 (18.4%)
Preexisting hepatitis (hepatitis B carrier)	3 (9.1%)
Indication for IVMP	
Active TED	26 (68.4%)
Compressive optic neuropathy	12 (31.6%)
Adjuvant therapy and subsequent surgery	
Methotrexate	26 (68.4%)
Radiotherapy	10 (26.3%)
Decompression surgery	8 (21.1%)
Mean duration of hyperthyroidism (SD), years	3.56 ± 5.76
Thyroid status	
Euthyroid	9 (23.7%)
Hyperthyroid	22 (57.9%)
Hypothyroid	6 (15.8%)

SD: standard deviation; IVMP: intravenous methylprednisolone; TED: thyroid eye disease.

**Table 2:** Outcomes.

	Pre-infusion	Post-infusion
Mean VA, right (SD)/log⁡Mar^a^	0.28 (0.29)	0.16 (0.26)
Mean VA, left (SD)/log⁡Mar^a^	0.27 (0.28)	0.17 (0.26)
Mean colours, right (SD)^a,b^	10.5 (3.6)	13.4 (5.4)
Mean colours, left (SD)^a,b^	13.1 (4.2)	13.9 (3.9)
Mean VISA inflammatory score (SD)	4.29 (1.14)	1.92 (1.12)

VA: visual acuity; SD: standard deviation.

^a^Only those with compressive optic neuropathy were analyzed.

^b^Colour vision was assessed using 15 plates of Ishihara colour vision test.

**Table 3:** Side effects (out of 242 infusions).

Side effects	*N*	%
Bradycardia <50 bpm	6	2.5
Hypertension SBP >160 mmHg and/or DBP >100 mmHg	12	5
Urticarial Rash-IVMP stopped	1	0.4
Nonspecific rashes or flushing^a^	5	2.1
Palpitations^a^	2	0.8
Shortness of breath^a^	2	0.8
Chest tightness^a^	1	0.4
Headache^a^	6	2.5
Insomnia^a^	2	0.8
Fever^a^	1	0.4
Gastritis^a^	1	0.4
Changes in LFT^b^	0	0
Changes in ECG	0	0

SBP: systolic blood pressure, DBP: diastolic blood pressure, IVMP: intravenous methylprednisolone, LFT: liver function test, and ECG: electrocardiogram.

^a^Documented from questionnaire after end of each infusion at 90 min.

^b^Defined as above, 3 times of upper limit of normal.
